# Precision approaches to paediatric hypertension: linking pathophysiology to therapy

**DOI:** 10.1007/s00467-025-07100-w

**Published:** 2025-12-19

**Authors:** Emily Haseler, Manish D. Sinha

**Affiliations:** 1 Department of Paediatric Nephrology, Evelina London Children’s Hospital, Guys & St Thomas NHS Foundation Trust, Westminster Bridge Road, London, SE1 7EH UK; 2 Department of Clinical Pharmacology, School of Cardiovascular and Metabolic Medicine, King’s College London, London, UK

**Keywords:** Hypertension, Children and young people, Cardiovascular risk, Obesity, Haemodynamics, Antihypertensive treatment, Personalised medicine

## Abstract

Blood pressure is the product of a complex interplay between the pumping action of the heart and the resistance capacity of both large and small arteries. These are modulated by several physiological processes, including the autonomic nervous system, renin angiotensin aldosterone system, neurohormonal influences and fluid balance. Dysregulation of one or more of these can lead to hypertension. Consideration of the key concepts underlying the generation and regulation of blood pressure is important to consider when selecting an appropriate antihypertensive agent. This review will outline the main physiological processes contributing to blood pressure and discuss their relevance to the pathophysiology and treatment of primary hypertension in children and young people.

**Graphical Abstract:**

**Figure:**
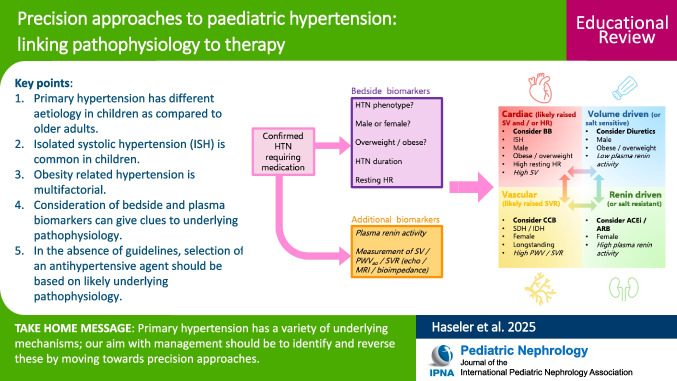
A higher resolution version of the Graphical abstract is available as Supplementary information.

**Supplementary Information:**

The online version contains supplementary material available at 10.1007/s00467-025-07100-w.

## Introduction

Hypertension in children is defined as repeated blood pressure (BP) measurements at or above the 95th centile for age, sex and height (or '≥' the regional threshold for the diagnosis of hypertension in adults), preferably including out-of-office measurements. Hypertension has increased in prevalence in children and adolescents over the last few decades. A recent meta-analysis reported a pooled prevalence of 4.0% (95% CI 3.29–4.78%), with a 75% increase in reported prevalence between 2000 and 2015 [1]. This is in large part due to the rising prevalence of primary hypertension (PH) in young people. PH is defined as hypertension for which no secondary cause can be found, and its increased prevalence is strongly linked to the parallel clinical epidemic of obesity. Emerging data from large population cohorts support the long-held assumption that hypertension diagnosed in childhood is associated with increased rates of major adverse cardiovascular events (MACE) in adulthood. Analysis of ~ 40,000 participants from the international Cardiovascular Cohort Consortium study found a hazard ratio of 2.0 (95% CI 1.2–3.4) for fatal MACE after a mean of 35 years of follow-up in individuals who were hypertensive as children [2].

Taken together, this evidence paints an alarming picture of increasing cardiovascular risk burden in the adolescent and young adult population, leading to increased cardiovascular risk burden in middle age over the coming decades. Despite this, relatively little is known about the pathophysiology of PH in children and in particular which antihypertensive agents are most effective in this population. The pathophysiology of hypertension was recently covered in an excellent review in this journal [3], and the focus of this current review will be to expand on key haemodynamic concepts and discuss their potential implications for treatment.

## Physiology of blood pressure: a multifactorial perspective

Blood pressure is an umbrella term referring to the pressure exerted on the walls of the blood vessels (arteries) within the circulatory system. Unless otherwise specified, it is assumed to refer to the systolic (peak) and diastolic (trough) pressures within the brachial artery, where it is most commonly measured with a sphygmomanometer. Blood pressure is the product of a complex interplay between the pumping action of the heart and the resistance capacity of both large and small arteries. These are modulated by a number of physiological processes, including the autonomic nervous system, renin angiotensin aldosterone system (RAAS), neurohormonal influences and fluid balance. Dysregulation of one or more of these can lead to hypertension. In addition, a multitude of modifiable and unmodifiable factors contribute to long-term modulation of BP, including (but not limited to) genetic and epigenetic factors, intrauterine and neonatal environment, salt intake, body habitus and chronic stress states.

**Fig. 1:**
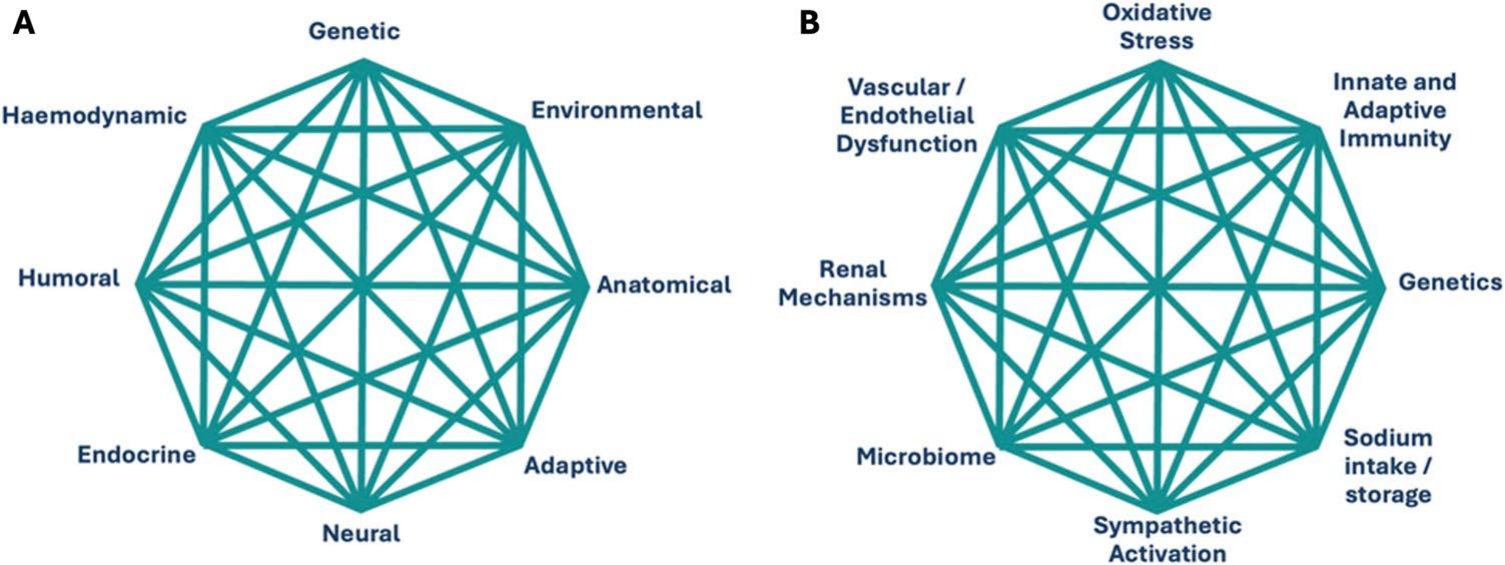
Original (**A**) and recently proposed update (**B**) for Page’s mosaic model of hypertension pathogenesis adapted from Page (1949) and Harrison et al. (2021)

These concepts were succinctly summarised by Page in his 1949 Mosaic Theory, who proposed eight “nodes” or “control centres” underpinning hypertension (Fig. 1A) [4]. Updates to this original Mosaic theory have been periodically published since, adapting the original eight concepts to the more specific physiological influences underlying most current research in hypertension (Fig. 1B) [5]. However, the central tenet of this theory remains unchanged. Hypertension arises from a unique “mosaic” of these driving factors in everyone, and the interplay between them forms major feedforward mechanisms resulting in its pathogenesis. Thus, PH in children is unlikely to arise from a single causative factor for each hypertensive young person. Current evidence regarding which processes predominate in the earliest stages of the development of hypertension is limited and not conclusive, but certain themes can be observed and are discussed further next.

## Static vs. pulsatile BP

Blood pressure can be separated into steady state or “static” and “pulsatile” or “dynamic” components, reflective of the haemodynamic determinants of mean arterial pressure (MAP) and pulse pressure (PP) respectively. This is of relevance in young people as elevation in PP alone leads to isolated systolic hypertension (ISH), a common presenting hypertensive phenotype in the young [6]. Elevation in MAP, in contrast, leads to isolated diastolic or systo-diastolic hypertension (SDH) as MAP is a closer approximation to diastolic BP (DBP). The same value of MAP may be derived from very different combinations of systolic BP (SBP) and DBP. For example, a MAP of 90 mmHg may be derived from values of 110/80 mmHg or 140/65 mmHg. Considering the determinants of each of these separately can shed insight into the haemodynamic origins of hypertension and hence better identify treatment targets.

### Static BP

Steady state or “static” BP refers to the constant perfusion pressure to the organs, quantified by MAP. The physiological processes contributing to static BP are summarised below.

#### Cardiac output (CO)

Cardiac output (CO) describes the volume of blood pumped by the left ventricle over a minute (L/min) and comprises heart rate (HR) multiplied by stroke volume (SV) (CO = HR × SV).

- Heart rate is determined by the relative influences of sympathetic vs. parasympathetic nerve fibres at the sino-atrial node.
- Stroke volume describes the volume of blood ejected by the left ventricle each cardiac cycle. It is determined by the ventricular contractile force versus aortic pressure at the end of diastole, otherwise known as the afterload. In other words, the heart must generate a force of contraction which exceeds the pressure in the aorta to open the aortic valve and eject blood into the systemic arterial circulation.

#### Systemic vascular resistance (SVR)

Systemic vascular resistance (SVR) represents the opposition that the terminal arteries and arterioles (30–300 µm in diameter, collectively termed the “resistance vasculature”) present to blood flow within the systemic circulation. The concept of resistance is fundamental to understanding how blood flow is regulated: a higher resistance limits flow, whilst a lower resistance facilitates it. This relationship between flow, resistance and pressure was first quantified by Poiseuille in 1840. Combining Poiseuille’s work with Darcy’s law and recognizing that conductance (the ease of flow) is the inverse of resistance (1/R) gives us Poiseuille’s law for flow through a cylindrical tube:

1 $$Q=\left(P1-P2\right)K=\left(P1-P2\right)\pi r^4/8\eta L=\left(P1-P2\right)/R\Rightarrow R=8\eta L/\pi r^4$$

where *Q* = flow in the vessel, *P*1 = pressure at the start of the vessel, *P*2 = pressure at the end of the vessel, *K* = conductance, *R* = resistance, *r* = radius of the vessel, *η* = viscosity of the blood, *L* = length of the vessel and *π*: (pi) = a mathematical constant approximately equal to 3.14159.

As resistance is inversely proportional to the fourth power of the vessel radius ($R\propto 1/{r}^{4}$), even a small reduction in radius produces a large increase in resistance. Under constant perfusion pressure (${P}_{1}-{P}_{2}$), flow varies inversely with resistance ($Q\propto 1/R$). Thus, halving the vessel radius ($r_2=\frac12r_1$) increases the resistance 16-fold and results in a decrease in flow because of this relationship:

$$\frac{{Q}_{2}}{{Q}_{1}} ={\left(\frac{{\mathrm{r}}_{2}}{{r}_{1}}\right)}^{4}={\left(\frac{1}{2} \right)}^{4}= \frac{1}{16}$$

This means that only 6.25% of the original flow remains after the radius is halved, corresponding to a drop from 160 to 10 mL/min. This example highlights that small changes in radius produce disproportionately large increases in resistance, markedly decreasing flow. It is also useful to consider an alternate scenario where flow must be maintained constant in the face of small reductions in arteriolar radius (e.g. from vascular remodelling). In this case, the subsequent increased resistance necessitates a large increase in perfusion pressure to maintain a constant flow. For example, if arteriolar radius is reduced by 10% (${r}_{2}=0.9{r}_{1}$), resistance increases by a factor (1/0.9)^4^ $\approx$ 1.524. At a constant flow this raises the required MAP from 70 mmHg to 70 × 1.524 $\approx$ 106.7 mmHg. Thus, a modest 10% narrowing produces a ≈52% rise in perfusion pressure.

**Fig. 2:**
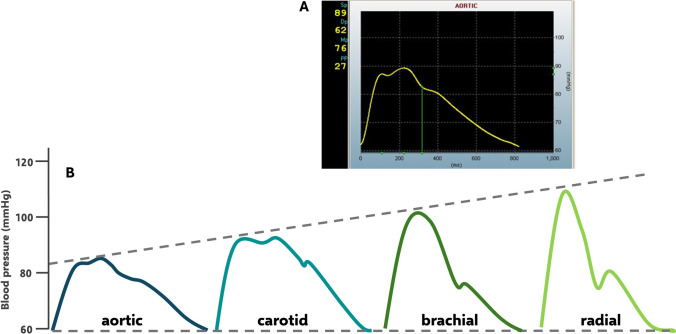
Systolic amplification in the arterial pulse wave An example of a derived aortic waveform following pulse wave analysis (PWA) measurement using direct tonometry (**A**). Amplification occurs as the pressure wave travels down the arterial tree from larger to smaller arteries e.g. from aortic to radial arteries (**B**), thus central SBP is lower than peripheral SBP. In these example waveforms from a healthy adolescent, aortic systolic pressure is ~ 85 mmHg and radial systolic pressure ~ 110 mmHg, giving a central systolic amplification of 25 mmHg

SVR is thus modulated by tiny fluctuations in the diameter of these “resistance” vessels, effected by the tone of their vascular smooth muscle. Though Poiseuille’s law refers to MAP rather than SBP, it also contributes to the effect of systolic pressure amplification as the arterial pulse wave travels distally through the arterial tree. Aortic or “central” SBP is typically around 20 mmHg lower in the larger, central arteries than in the smaller distal arteries. This can be measured with pulse wave analysis (PWA) as shown in Fig. 2 [7].

### Pulsatile BP

**Fig. 3:**
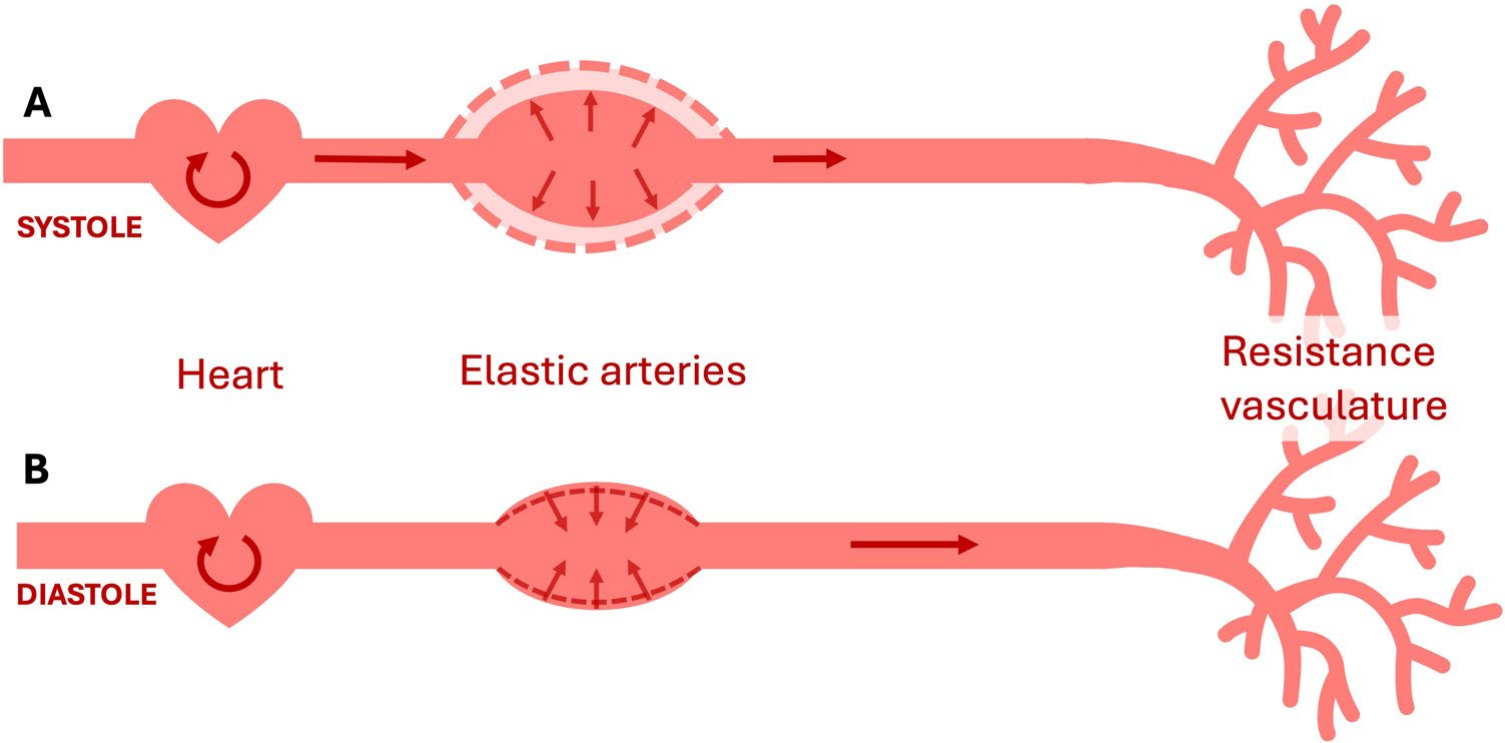
Windkessel model of circulation The large arteries (mainly aorta) act as capacitators, absorbing and storing pressure during systole in their elastic walls (**A**) and discharging it during diastole (**B**) resulting in a blunt peak of the aortic pressure wave

The concept of MAP as the product of CO and SVR and the determinant factors of these is well established and grounded in relatively simple laws of physics. Pulsatile haemodynamics refers to amplitude (i.e. PP) and shape of the arterial pressure wave propagated through the large arteries over a cardiac cycle. Its contributing factors are more elusive and controversial than those of “static” BP. These factors can be considered as the balance between left ventricular outflow dynamics and large artery compliance, similar to  the determinants of SV. It is helpful to consider the vascular system as containing resistor and capacitor elements (the 3 element Windkessel model) [8], summarised in Fig. 3.

#### Left ventricular outflow haemodynamics

SV and its determinants are the most recognised parameters contributing to left ventricular outflow; however, in recent years, attempts have been made to additionally quantify flow dynamics in the early phase of systole. This enables capture of not just the total flow over the cardiac cycle (synonymous with SV) but the amplitude and timing of the peak of the flow wave, which may approximate better with PP variability than SV alone. One such marker is first phase ejection fraction (EF1), a new biomarker describing the ejection fraction from the beginning of systole up to the point of maximal LV contraction. This has been found to be altered in young people with PH compared to healthy controls even in the absence of significant left ventricular remodelling [9]. Secondly, left ventricular outflow tract velocity has also been shown to be higher in hypertensive vs. normotensive young people [10].

#### Large artery stiffness

The elasticity of the large arteries, and in particular the proximal aorta, acts as a capacitor during the cardiac cycle. Some of the initial energy generated by the force of ventricular contraction is stored as elastic energy. Elastic recoil distributes some of this force later in the cardiac cycle to in effect “flatten” the pressure wave and reduce pulse pressure. This can be measured as distensibility, e.g. comparing the cross-sectional area of the vessel lumen at end systole compared to end diastole for any given pressure change. Large artery haemodynamics were recently reported from the SHIP AHOY study, in which SBP in a multi-ethnic adolescent cohort was stratified into low (< 80th centile), mid (80th–< 90th centile), and high (≥ 90th centile) groups. Aortic distensibility was lower in the mid and high groups compared to the low group [11].

Alternatively, and more commonly, the reciprocal of elasticity, stiffness, is measured. Pulse wave velocity (PWV) is the most widely accepted marker of arterial stiffness; a stiffer (less distensible) vessel wall can propagate the arterial pulse wave faster; therefore, PWV increases proportionally with wall stiffness. PWV can be measured relatively easily and has therefore become both a key functional marker of vascular hypertension mediated organ damage (HMOD) and a marker of interest in delineating large artery haemodynamics.

Frequently used sites to measure PWV are carotid-femoral or carotid-brachial. A recent systematic review of the published data shows higher PWV in hypertensive compared to normotensive children, although the heterogenous nature of the data limited the ability to perform meta-analysis [12]. In SHIP AHOY, carotid-femoral PWV was higher in the high (5.4 ± 0.9 m/s) compared to the low BP group (4.8 ± 0.7 m/s, *p* < 0.05) [11]. It should be noted that mean PWV increases more distally in the arterial tree: in adults, mean PWV is 4–5 m/s in the ascending aorta, 6–7 m/s in the descending aorta, and 8–9 m/s in the iliac and femoral arteries. Considering pulsatile haemodynamics, a proximal PWV measurement may be more relevant than PWV measured at more peripheral sites, e.g. the brachial artery [13]. Table 1 summarises the most used research methodologies to measure key haemodynamic parameters of PWV, PWA, CO, SV and SVR.

**Table 1:** Measurement of haemodynamics

Haemodynamic measurement technique *Example device*	Parameters measured and sites (PWV)	Advantages	Disadvantages	Validated in children?	Reference data in children	Other references
Applanation tonometry *SphygmoCor*	PWV*cf* PWA (central BP)	- Gold standard measurement of PWV - technique using direct measurement with pressure transducer	- Product support now withdrawn - Operator dependent and difficult technique to learn - Sequential measurement between sites vulnerable to heart rate changes during measurement	Noninvasive gold standard for PWV/PWA	Yes [70]	[71]
Hybrid applanation/oscillometric *SphygmoCor XCEL*	PWV*cf* PWA	- PWV: Simultaneous measurement at both sites (femoral = derived measurement from thigh cuff) - Mainly cuff based, less technical/operator dependent	- Few studies in children - Still some operator dependence and technical difficulty	Yes (PWV) [72]	No	
Pressure transducing cuffs/sensors *Vicorder (V)* *Complior (C)*	PWV*cf (V,C) OR bf (V)* PWA	- Simultaneous measurement at both sites - Low margin of error (V-bf) - Dual site measurement, but technically easier than applanation (V)	- Some technical expertise required (especially C)	Yes (V, PWV) [71]	Yes (PWV) [73]	
Osclillometric *Mobil-O-Graph (M), Arteriograph (A)*	PWV Aortic (indirect from brachial cuff) PWA (cBP) CO/SVR	- Easy to use - Low intra-operator variability - 24-h monitoring functionality	- Indirect measurement from single site - Measurements less comparable than between pressure transduction methods	Yes (PWV, cBP, M) [74, 75]	Yes (M) [73]	
Echocardiography	CO/SV	- Can be obtained from same protocol as clinical functional and structural cardiac assessment - Cheaper and more widely available than MRI	- Technical expertise needed - Intra observer variability	Yes	Yes [76]	[10]
Echocardiography/vascular ultrasound	PWV *Aortic (dual probe between two sites)*	- Measurement derived from intramural pressure: can manipulate to isolate BP independent effects of PWV	- Technically challenging and resource intensive (2 operators needed). Limited to small experimental research studies	No	Yes [77]	[78]
Cardiac MRI	CO/SV PWV *aortic*	- Can be obtained from same protocol as clinical functional and structural cardiac assessment - PWV: Measurement of peak flow transit time between any two points in aorta - Precise measurement with low intra operator variability - Standardisation of measurements and resource intensive technology continuing to improve with AI based analysis	- High cost - Heterogenous post processing algorithms to derive PWV from images – may limit comparability between studies	Non-invasive gold standard for PWV/PWA	Yes [79, 80]	[81]
Gas Rebreathe	CO	- Precise with low intra operator variability	- No studies in children outside congenital cardiac disease - Expensive equipment required, limited to research settings	No	No	[18]
Bioimpedence	CO/SVR	- Easy to use - Low intra-operator variability - Can be used during exercise/active children	- Indirect measurement based on algorithm/assumptions	No	No	[58]

*PWV* pulse wave velocity, *cf* carotid femoral, *PWA* pulse wave analysis, *BP* blood pressure, *V* Vicorder, *C* Complior, *bf* brachial femoral, *M* Mobil-O-Graph, *A* Arteriograph, *cBP* central BP, *CO* cardiac output, *SVR* systemic vascular resistance, *SV* stroke volume, *MRI* magnetic resonance imaging, *AI* artificial intelligence

Section summary:

- “Static” or steady state BP is represented by MAP, determined by SVR × CO.
- “Pulsatile” or dynamic BP is represented by PP, determined by the interaction between early ventricular ejection and proximal aortic stiffness.
- Key differences between static and pulsatile BP are summarised in Table 2.

**Table 2:** Key distinctions between static and pulsatile BP: haemodynamic determinants, clinical correlates and associations

	Static blood pressure	Pulsatile blood pressure
Key haemodynamic parameter	MAP	PP
How is it measured?	CO × SVR	No mathematical equation universally accepted, aortic (central) PP proposed to be derived from *PP* = *η* × *PWV*_*ao*_ × *U* [82]
Determinants	CO (HR × SV), SVR	Complex interplay between proximal aortic stiffness and left ventricular ejection haemodynamics
Correlating hypertensive phenotype	• SDH, IDH	• ISH
Clinical associations	• Longstanding hypertension • Middle age • Female • Secondary causes	• Childhood hypertension • Male • Obesity
Cardiovascular risk	• Lower long term cardiovascular risk in young adults with IDH than SDH	• Lower long term cardiovascular risk in young adults with ISH than SDH • Some evidence of progression from ISH to SDH over time [83]

*MAP* mean arterial pressure, *PP* pulse pressure, *CO* cardiac output, *SVR* systemic vascular resistance, *η *viscosity of blood, *PWV*_*ao*_ pulse wave velocity in proximal aorta, *U* aortic blood flow velocity, *HR* heart rate, *SV* stroke volume, *SDH* systodiastolic hypertension, *IDH* isolated diastolic hypertension, *ISH* isolated systolic hypertension

## Abnormal static vs. pulsatile BP may reflect underlying pathology

Two key processes contributing to and maintaining hypertension in adults are thought to be age-related vascular stiffening (arteriosclerosis) and hypertension-related vascular remodelling. Vascular remodelling occurs throughout the arterial tree in response to hypertension-induced shear stress and stretch-related damage. In small, muscular arteries, the resulting stiffening of the vessel wall causes increased SVR, which disproportionately increases MAP rather than PP. This increased MAP is reflected by the clinical picture of SDH, the predominant hypertensive phenotype of middle age.

**Fig. 4:**
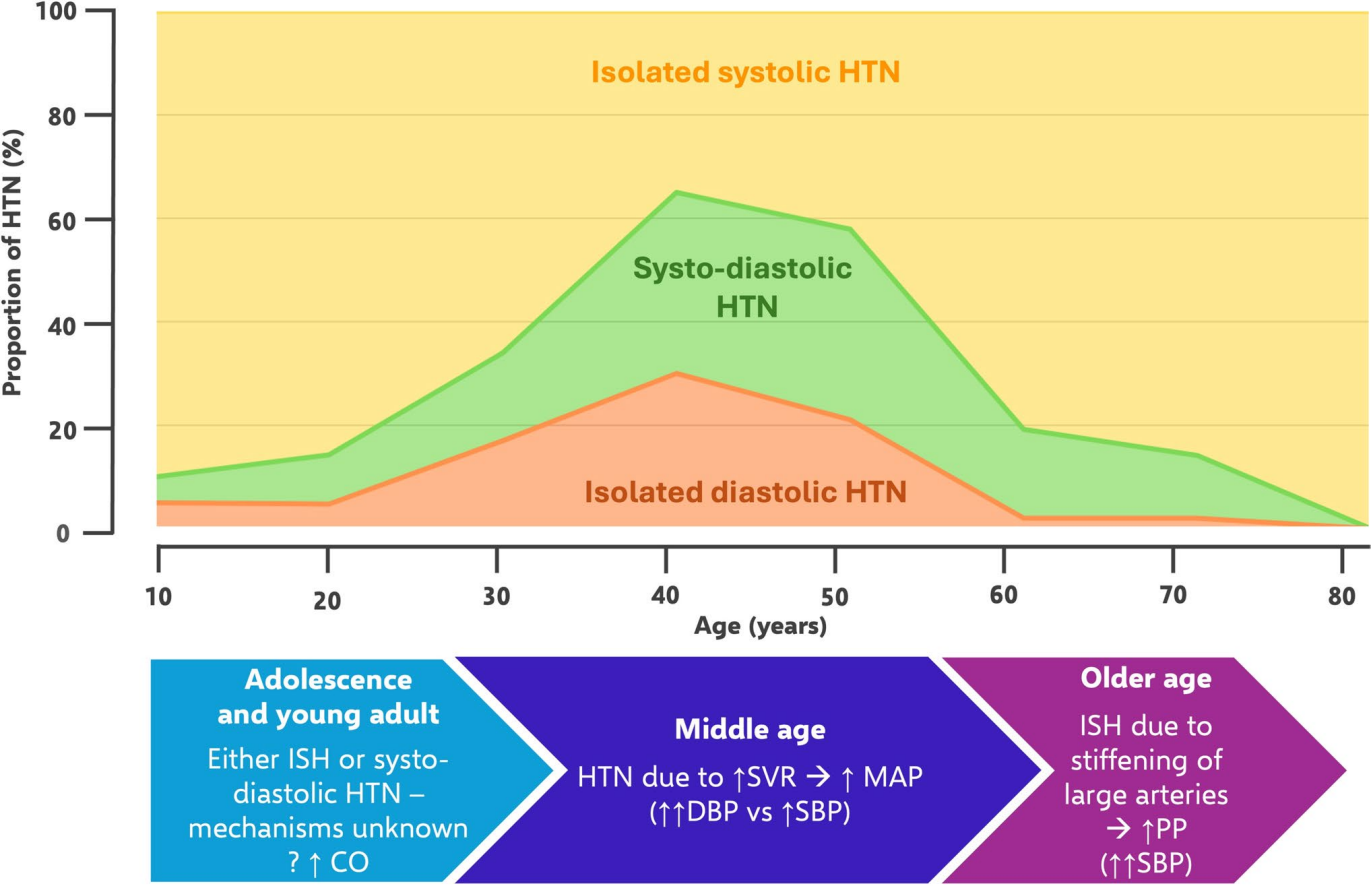
Schematic diagram showing the prevalence of different hypertension phenotypes across the age range (top) with potential underlying aetiological mechanisms (bottom) adapted from published literature [6, 69]. HTN, hypertension; ISH, isolated systolic hypertension; CO, cardiac output; SVR, systemic vascular resistance; MAP, mean arterial pressure; DBP, diastolic blood pressure; SBP, systolic blood pressure; PP, pulse pressure

Later in life, arteriosclerosis begins to affect large (predominantly elastic) arteries, resulting in decreased compliance through the cardiac cycle and in turn producing a greater PP for any given SV. This increasing PP presents as ISH, the predominant hypertension subtype in older adults, especially women [14]. As previously mentioned, ISH also predominates in children; however, these patients are otherwise well. Age-related arteriosclerosis is therefore unlikely to play a key aetiological role. Although PWV has been shown to be elevated in children with PH compared to healthy controls, mean PWV remains far below the threshold which in adults is felt to represent pathological vascular HMOD [12]. The changing phenotypes of hypertension over the lifespan with relation to proposed pathogenesis are shown in Fig. 4. This has led to efforts over the past decades to investigate other pathophysiological mechanisms underlying hypertension in children and young people, with the aim of better targeting prevention and treatment of hypertension in this group.

### “Cardiac”-driven hypertension

From small observational studies in the 1960s, a “cardiac” driven phenotype of disproportionately raised cardiac output in comparison to systemic vascular resistance has been proposed as a key mechanism driving PH in young people. This was quickly identified as likely due to autonomic nervous system dysfunction. In a preselected high CO group of young hypertensive patients, infusion of propranolol (i.e. selective beta receptor blockade) reduced CO and HR more so in the hypertensive group than in normotensive controls [15]. The residual differences between the two groups were eliminated following subsequent atropine infusion. This would suggest that both sympathetic nervous system (SNS) overactivity and reduced parasympathetic system activity (i.e. vagal tone) are responsible for the haemodynamic differences seen in these “hyperdynamic” patients [16].

Early astute observations by investigators reported a positive correlation between BP, HR, and measures of adiposity or progressively higher HR in those with higher BPs and higher body mass index (BMI) [6, 17]. These investigators suggested that the HR differences, coupled with increased BP variability in obese compared to normal weight participants, were indirect evidence of SNS involvement [6]. Population studies in young adults further support the hyperdynamic state theory, the most notable of which is from the ENIGMA cohort in the UK. Investigators studied 1008 predominantly male university students with a mean age of 20 and found higher CO (as a result of raised SV) but not higher SVR in those with ISH compared to normotensive individuals [18].

Obrycki and colleagues observed a stepwise increase in CO and SV from normotensive through pre hypertension into hypertension (*n *= 66, *n *= 30 and *n *= 92 respectively), but no increase in HR or SVR [19]. The hypertensive group was predominantly male, with a high proportion of obesity (~ 30%) and ISH (60%). The same investigators then reported on a group of 48 patients found to have spurious hypertension, a term used to describe those with raised peripheral SBP (ISH) but normal central BP. Re-evaluation of the 48 children with spurious hypertension following 12 months of non-pharmacological intervention showed 26% had no change in phenotype, 23% had developed true hypertension (elevated peripheral and central BP), and the rest had become normotensive [20]. Despite no change in BMI, those who had developed true hypertension were found to have a significant increase in CO (SV more so than HR) but no increase in SVR.

Li and colleagues attempted to comprehensively characterise the static and pulsatile BP haemodynamics in 31 hypertensive patients, predominantly boys, mean age 15 years, of whom 2/3 were on antihypertensive medication. Components of both “static” and “dynamic” BP were compared to age-matched normotensive controls [10]. They reported that those with hypertension had higher CO, SV, and HR compared to normotensive controls, with no difference in SVR. In addition, regarding dynamic BP, hypertensive participants had higher flow and volume velocities across the left ventricular outflow tract and higher proximal aortic PWV. These differences were proportional to their increased pulse pressure of 40% compared to controls. Li et al. have suggested increased SNS activity as the pathophysiological link between obesity, PH, increased CO, and altered ventricular ejection dynamics [21, 22].

### “Vascular”-driven hypertension

Not all evidence in the young is in favour of an initial “cardiac” driven pathophysiology. Interestingly, in the ENIGMA study, individuals with SDH had raised HR and SVR, but normal SV and CO compared with normotensive individuals [18]. In a second analysis at the slightly older age of 25 years, hypertensive males displayed a “cardiac” haemodynamic phenotype with increased CO, SV and HR compared to normotensive controls. Whilst hypertensive females also had raised CO, they otherwise displayed a “vascular” phenotype with increased SVR and aortic stiffness compared to normotensive controls [23]. In a younger study cohort aged 17 years, Park et al. reported on longitudinal data from the ALSPAC study. They found increased HR, SV and SVR in the highest quintile of SBP, with proportionate contributions of these haemodynamic properties to all BP quintiles. Their analysis did not though comment on the potential influence of body size or obesity on these results [24].

Lastly, a smaller subset of hypertensive patients displays isolated diastolic hypertension (IDH). This is the most uncommon subtype in children and was described recently in a large cohort from the NHANES study in the US. Individuals with IDH were found to have a low prevalence of overweight/obesity, to be more likely to be White, female, and to have higher resting heart rates [25]. Whilst this cohort did not have other haemodynamic parameters measured (CO, SV, and SVR), the findings are consistent with those of ENIGMA in the UK. The raised HR seen in these “vascular” patterns of hypertension is intriguing as it implies a different type of dysfunction in these patients with IDH than those with ISH and a high SV. Raised HR is worth considering in particular detail due to the known correlation between raised resting heart rate and increased risk of MACE in adult studies [26]. However, this has yet to be explored in longitudinal paediatric cohorts.

These data suggest that at the earliest stages of hypertension, distinct haemodynamic phenotypes may be present. Young people with ISH are more likely to have a “cardiac” driven hypertension characterised by raised SV, whereas those with SDH are likely to have a vascular component to their hypertension characterised by raised SVR. It remains interesting to consider that over time, uncontrolled “cardiac” driven hypertension will lead to vascular remodelling, an increase in SVR, followed by an increasing MAP and a shift from ISH to SDH [22, 27]. Further research should focus on the role of elevated HR in childhood and its associations with different hypertensive phenotypes. As a key determinant of CO, in small studies elevated HR is associated with elevated SV in hypertensive CYP displaying a “cardiac” phenotype. However, in larger population studies elevated HR appears to cluster with elevated SVR and demographic features more suggestive of a “vascular” phenotype.

Section summary:

- “Cardiac” hypertension refers to that where elevated BP is associated with elevated SV and hence CO.
- “Vascular” hypertension refers to that where the primary haemodynamic disturbance is raised SVR.
- Published evidence regarding elevated HR suggests contribution to both “cardiac” and “vascular” phenotypes of hypertension in children and young people and need further careful consideration especially at the earliest stages.
- “Cardiac” hypertension has been associated with ISH, the predominant PH phenotype in young people. “Vascular” hypertension has been associated with SDH, whose prevalence increases into middle age.

## Role of salt sensitivity

Salt and volume handling are fundamental to blood pressure regulation, as first proposed by Guyton over 50 years ago [28]. Impaired salt and water handling is a key mechanism underlying hypertension in kidney impairment, and most mutations in monogenic hypertension involve sodium handling pathways [29]. Data from adults and children strongly support individual variation in salt handling [30]. Research studies have sought to categorise individuals by their response to salt using a salt sensitivity test involving a period of high salt intake followed by a period of low salt intake. Individuals who demonstrate a significant drop in BP (8–10 mmHg for hypertensives and 3–5 mmHg for normotensives) are labelled “salt sensitive” (SS) as compared to “salt resistant” (SR) individuals who do not [31]. Despite variability in protocols and definitions, efforts towards consensus are emerging [31]. Although unlikely to be assessed routinely in clinical practice, the concept informs mechanistic understanding and has been proposed as a strategy for individualised therapy.

Historically, SS has been attributed to an impaired or blunted natriuretic response to a salt load, resulting in sodium and water retention and transient increases in CO before the sodium load is slowly excreted. Over time, there is a progressive upward shift of the “set point” for BP and the volume loaded state becomes the new normal. In contrast, SR individuals excrete salt and water with minimal volume or haemodynamic shifts and experience limited BP drop following a salt sensitivity test. More recently, impaired vasodilatory capacity of the renal and systemic vasculature in response to salt loading has been implicated. This leads to both inappropriately increased SVR and downstream effects on renal salt handling, contributing to a prolonged increase in BP [32].

Adult studies indicate a higher prevalence of salt sensitivity among Black individuals, females, and those of increasing age. There is also evidence to suggest variability in salt sensitivity among young individuals, indicating differential responses to salt loading. In the few studies in which SS has been formally tested, SS prevalence in unselected patient groups ranges from 12–40%, though this prevalence may be higher in cohorts of hypertensive young people [30, 33].

The strong association between SS and Black ethnicity has been repeatedly demonstrated, including a young adult study reporting 18.4% prevalence in White versus 37.3% in Black participants [33]. However, more recent data highlight significant variations both between and within ethnicities, reflecting environmental factors, the social construct of race, and diverse genetic ancestry [34]. Whilst tailoring treatment to ethnicity may currently be a useful tool given the current evidence base, future advances in pharmacogenomics may enable more precise tailoring. For example, variants in epithelial sodium channel pathways have been linked to altered drug responses in Black cohorts [35]. In pooled analyses of nearly 5000 participants, six gene variants affecting sodium and aldosterone handling were associated with differential responses to candesartan and hydrochlorothiazide, with per-allele effects up to ± 3.5 mmHg between Black and White participants [35].

Section summary:

- SS is a phenotypic variant of hypertension in which blunted response to salt load leads over time to an increased set point for BP.
- It is likely to affect up to 40% of young people with PH and in adults has been associated with Black ethnicity and female sex.

## Obesity-related mechanisms are key

Obesity is well established as an independent risk factor for hypertension. Prevalence of hypertension in those with overweight and obesity has been estimated between 15 and 40%, depending on the definition and number of measurements used [1, 6]. A meta-analysis including over 8000 children found SBP to be 7.5 (SD 3.4 to 11.6) mmHg and DBP 4.1 (2.1 to 6.1) mmHg higher in obese compared to normal-weight children [36].

There are multiple potential mechanisms contributing to hypertension in obesity, reviewed in detail elsewhere [21, 37]. Obesity leads to a pro-inflammatory state established by excess adipose tissue, in particular visceral as opposed to subcutaneous adipose tissue [21]. Hyperinsulinaemia is known to result in SNS activation, and several cross-sectional and epidemiological studies have observed higher HR, SV or CO in those with excess weight [6, 38]. Obesity has also been linked to SS states in children, with reversibility of this phenotype following weight loss [39]. Lastly, adipose tissue has been shown to release angiotensin II and aldosterone, directly linking obesity with RAAS activation [40].

Conversely, vascular factors are less likely to contribute to early hypertension in children with excess weight. Interesting findings from the ALSPAC longitudinal cohort showed slightly lower PWV in obese compared to normal weight children at the age of 10 years. However, in a smaller subset, persistently increased fat mass over the subsequent 7 years was associated with an increased PWV at 17 years compared to those who had a persistently low or reducing fat mass [38, 41]. Lurbe and colleagues similarly observed lower PWV with increasing weight category in a population sample of 500 children aged 8–18 years [42]. Further, Stabouli and colleagues observed increased PWV in the presence of both hypertension and obesity, but that BMI alone was not a determinant of PWV [43].

These findings suggest the pathophysiology of obesity-related hypertension is more likely to be initiated by multiple mechanisms linked to volume and hyperdynamic circulation rather than vascular remodelling. However, since excess weight is associated with earlier onset hypertension and accelerated vascular ageing, it is also likely that vascular factors start to contribute to hypertension earlier in the life course than in patients with normal weight [37].

Section summary:

- Obesity is prevalent in childhood hypertension with multifactorial pathophysiological mechanisms linking the two.
- These include “cardiac” haemodynamics, activation of the RAAS and promotion of SS state.

## Implications for treatment

There is a lack of high-quality data available to guide pharmacological management in children and adolescents with hypertension. This gap extends to young adults, with most large, randomised controlled trials (RCTs) including participants with mean ages of 40 years and above. As discussed above, these trials probably lack applicability to the younger age group given their different underlying pathophysiology.

Existing studies comparing effects of different antihypertensive agents in paediatrics and young adults are mainly industry-funded and either placebo controlled or comparing two agents with similar mechanisms of action. Two meta-analyses over the last decade have been unable to draw conclusions regarding the superiority of one family of antihypertensives compared to another, either for hypertensive young people in general or selected groups [44, 45].

A small RCT comparing nebivolol vs. ramipril in young men aged 16–24 years with hypertension found a comparable BP difference between the groups following 12 weeks of treatment. However, ramipril treatment was associated with improvements in Stiffness Index and in metabolic profile including glucose and high-density lipoprotein (HDL), whereas nebivolol led to BP reduction in the absence of any additional benefits [46]. Another interesting pilot RCT from 1983 compared centrally acting α adrenergic agonist clonidine with hydrochlorothiazide in 30 adolescent patients. Both agents significantly reduced SBP, but clonidine additionally reduced DBP, heart rate, and plasma catecholamines. Following a mental stress test, clonidine-treated patients had a blunted increase in DBP and HR, implying some element of central autonomic dysfunction underlying their hypertension [47].

Samuel and colleagues have explored “n of 1” study design in two innovative publications. In the first, a small crossover study rotated 32 adolescent participants through amlodipine, lisinopril, and hydrochlorothiazide monotherapy and evaluated efficacy using ambulatory BP monitoring (ABPM). Lisinopril was the most effective and tolerated medication in half the participants, followed by amlodipine, then hydrochlorothiazide. Interestingly, “preferred” medication was not predicted by sex, BMI, or starting BP, and the majority (67%) of African American participants preferred lisinopril. The same group then followed with a small RCT comparing usual care with the “*n* of 1” approach. They found a modest increase in the likelihood of achieving BP control after 6 months using the “n of 1” approach in which patients cycled through two antihypertensive agents (amlodipine and lisinopril for most participants). These indicate promising results of an individualised approach to patient care and of protocolised titration to ABPM. However, it should be noted that this resource-intensive approach may not be accessible in all healthcare settings [48, 49].

Currently, both the European Society of Hypertension (ESH) and American Academy of Pediatrics (AAP) guidelines specify that the choice of treatment should address the pathophysiology underlying the cause of hypertension as far as possible [50, 51]. However, except for the use of angiotensin converting enzyme inhibitor (ACEi)/angiotensin receptor blocker (ARB) agents in chronic kidney disease, this is largely a pragmatic rather than evidence-based approach [52]. Table 3 details paediatric studies where subgroup analysis has been performed looking at the responsiveness of demographics to pharmacological treatment. Below are discussed some additional treatment considerations based on the pathophysiological mechanisms underlying paediatric hypertension described in this review.

**Table 3:** Summary of studies evaluating treatment of primary hypertension in children in which efficacy has been considered in subgroups

Medication class	Study	Design	Medications	Findings	Specific groups
ACEi	Li (2004) [84] Menon (2006) [85]	Multicentre, multinational placebo withdrawal RCT *n *= 253, 6–16 years Post hoc analysis, *n *= 52 Black vs. 152 White	Fosinopril (0.1 mg/kg/0.3 mg/kg/0.6 mg/kg) → placebo vs. fosinopril	Fosinopril well tolerated and effective No dose response seen in main analysis	Black individuals did exhibit dose response between low and medium/high doses. No difference within each ethnicity regarding sex, BMI or Tanner stage
	Soffer (2003) [86]	Multicentre placebo withdrawal RCT *n *= 115, 6–16 years	Lisinopril (1.25 mg/5 mg/40 mg) → placebo vs. lisinopril	Lisinopril well tolerated and effective Dose response relationship seen	Dose response similar across all subgroups: Sex (male, female) Age (≤ 12, > 12 years old) Weight (< 50 vs. ≥50 kg) Tanner stage (≤ 3, > 3) Ethnicity (White, African American, Hispanic, Asian) Region (USA, non-USA)
	Wells (2002) [87]	Multicentre, multinational placebo withdrawal RCT *n *= 110, 6–16 years	Enalapril (1.25 mg/5 mg/40 mg) → placebo vs. enalapril	Enalapril well tolerated and effective Dose response relationship seen	Similar efficacy all subgroups: Sex (male, female) Age (≤ 12, > 12 years old) Tanner stage (≤ 3, > 3) Ethnicity (White, Black, Other) Region (USA, non-USA) However, trend seen for more pronounced DBP drop in non-White vs. White ethnicities
ARB	Wells (2011) [88] Meyers (2011) [89]	Multicentre, multinational placebo withdrawal RCT *n *= 261, 6–16 years Post hoc analysis *n *= 142 obese vs. 119 non obese	Valsartan (20 mg/80 mg/160 mg) → placebo vs. valsartan	Valsartan well tolerated and effective	Similar efficacy all subgroups: Sex (male vs. female) Age (≤11 vs. > 11 years) Weight (< 35 vs. ≥35 kg) Tanner stage (< 3 vs. ≥3) Ethnicity (Black vs. White) Region (USA vs. non-USA) Obese vs. non obese patients
	Hazan (2010) [90]	Multicentre placebo withdrawal RCT Cohort A (multiethnic): *n *= 190 Cohort B (black): *n *= 112	Olmesartan (5 mg/40 mg) → placebo vs. olmesartan	Olmesartan well tolerated and effective at lowering SBP and DBP, significant withdrawal effect with high dose only	Smaller magnitude of effect in Black vs. multiethnic cohorts (difference not statistically significant)
	Shahinfar (2005) [91]	Multicentre placebo withdrawal RCT *n *= 175, 6–16 years	Losartan (5 mg/50 mg/100 mg) → placebo vs. losartan	Losartan well tolerated and effective	Dose response not statistically significant between: Sex (male vs. female) Age (≤12 vs. > 12 years) Tanner stage (≤3 vs. > 3) Ethnicity (Black vs. White vs. Hispanic vs. Others) However, trend seen for more pronounced dose response in White vs. non-White ethnicities
Beta-blocker	Batisky (2007) [92]	Multicentre placebo controlled RCT *n *= 140, 6–16 years	Metoprolol (0.2 mg/kg/1 mg/kg/2 mg/kg) vs. placebo	Metoprolol well tolerated and effective Dose response relationship seen for DBP	BP changes independent of: Sex (male vs. female) Age (≤11 vs. > 11 years) Weight (< 35 vs. ≥35 kg) Tanner stage (< 3 vs. ≥3) Ethnicity (Black vs. White) Overweight patients had less pronounced SBP reductions
Calcium channel antagonist	Flynn (2004) [93]	Multicentre, multinational placebo withdrawal RCT *n *= 268, 6–16 years	Amlodipine (2.5 mg/5 mg) → placebo vs. amlodipine	Amlodipine well tolerated and effective Dose response relationship seen	Similar efficacy in subgroups by ethnicity Greater SBP and DBP reduction in females compared to males No significant change in heart rate
	Trachtman (2003) [94]	Multicentre placebo controlled RCT *n *= 133, 6–16 years	Felodipine (2.5 mg/5 mg/10 mg) vs. placebo	Felodipine safe and well tolerated Main analysis showed DBP reduction in medium dose (5 mg) group only, not in 2.5 mg or 10 mg groups	Subgroups showed significantly higher BP reduction in younger/Tanner stage ≤3 participants compared to older/later puberty and in males compared to females No difference between ethnicities (Black vs. non-Black)
Combinations/multiple monotherapy agents	Sorof (2003) [95]	Multicentre, multinational, placebo controlled RCT *n *= 94, 6–17 years	B/HT vs. placebo B: titrated to effect 2.5 mg/5 mg/10 mg HT: 6.25 mg	B/HT safe and well tolerated. Modest SBP/DBP reduction compared to placebo but % reaching target BP not different between treatment and placebo groups	Significant BP reduction (both SBP and DBP) only seen in: - Younger children 6–12 years - Higher baseline SBP > 5 mmHg above 95th centile
	Berenson (1990) [96]	Single centre RCT, ethnicity, height and sex matched treatment/control pairs *n *= 95, 8–18 years	Treatment:P/CT plus education programme vs. no intervention	P/CT safe and well tolerated. Persistent drop in SBP/DBP of 4.4/2.7 mmHg in treatment vs. observation group	No statistically significant differences between sex and ethnicity Trend towards larger response in Black girls SBP change determined by HR change in White participants and weight change in Black participants

*ACEi* angiotensin converting enzyme inhibitor, *RCT* randomized controlled trial, *BMI* body mass index, *USA* United States of America, *DBP* diastolic blood pressure, *ARB* angiotensin receptor blocker, *SBP* systolic blood pressure, *B/HT* Bisoprolol/Hydrochloro-thiazide, *P/CT* propranolol/chlorthalidone, *HR* heart rate

### Renin-directed therapy

Plasma renin has long been proposed as a potential biomarker to help guide antihypertensive choice or predict response to treatment, based on its potential ability to distinguish SS from SR hypertension. Low renin states have been postulated to correlate with volume-mediated (SS) hypertension, which in turn may respond better to diuretics and vascular-acting agents, e.g. calcium channel blocker (CCB). Conversely, high renin states are postulated to correlate with renin-mediated (SR) hypertension, which may be more likely to respond to RAAS inhibitors and β-blockers.

These observations and their association with age and ethnicity in various clinical trials have informed guidelines on the choice of antihypertensive agent. The National Institute for Health and Care Excellence (NICE) in the UK recommends CCB as first-line in those over 55 years or patients of any age from Black African or African-Caribbean family origin. Conversely, RAAS blockade is recommended as first-line in patients under 55 years of age from any other ethnicity [53].

Laragh proposed a formal stratification of hypertensive adult patients by plasma renin activity (PRA). He described volume-mediated (synonymous with SS) hypertension as characterised by a very low plasma renin activity (< 0.65 ng/ml/hr). In contrast, patients with PRA above this threshold were classified as having “renin vasoconstrictor” hypertension (synonymous with SR) [54].

Whilst this has not been prospectively evaluated in any large-scale clinical trials, studies in adults have reported on the predictive value or correlation of PRA with BP response to different classes of antihypertensive medication [55]. Laragh’s group published one small RCT comparing renin-guided therapy to usual care and reported a significantly lower SBP in those receiving renin-guided therapy, with no improvement in DBP compared to usual care [56]. A multicentre study in Africa (Kenya, South Africa, and Nigeria) used ELISA-based point-of-care testing to profile both renin and aldosterone and found improvement in both SBP and DBP. Notably, treatment of low renin hypertension was either amiloride or potassium-sparing diuretic depending on aldosterone status [57].

Barriers to the implementation of this method in the paediatric population include equity of access to renin testing, although note the point-of-care testing used in the study above [57]. In addition, existing antihypertensive treatment may lead to difficulty in interpreting renin values, although this has been comprehensively considered by its proponents [54]. Lastly, low renin hypertension may be due to a specific cause (primary aldosteronism, monogenic Liddle syndrome variants), which should be considered and investigated prior to proceeding with a renin-directed approach to PH. As discussed above, there is no evidence base to support its use in the treatment of PH in children, although a renin-based approach may be a candidate for consideration in future clinical trials.

In the meantime, it would seem logical to avoid use of RAAS blockers as a first-line agent in the context of very low plasma renin levels, as hypertension is more likely to be volume-driven. In this context, a CCB or diuretic should be considered, and a low-salt diet reinforced. Additionally, consider using 24 h urine sodium excretion as a guide for baseline and follow-up salt intake.

### Haemodynamic-directed therapy

Another potential framework to personalise hypertension therapy may be around haemodynamic status, with the aim of not only normalising BP but also the specific haemodynamic abnormality contributing to high blood pressure, i.e. increased SVR, SV, or HR. This may be additionally desirable in the paediatric population as these patients are in the early stages of hypertension with, presumably, fewer simultaneous pathophysiological processes contributing to their raised BP. Correction of the single isolated abnormality may not only lower BP but also prevent or delay progression to irreversible pathophysiology through, for example, vascular remodelling. This has been explored in adults using direct measurement of haemodynamics with bioimpedance, with these results used to personalise therapy. For example, a moderately sized RCT comparing a treatment titrated to bioimpedance haemodynamics found a 6/7 mmHg additive benefit on SBP and DBP reduction from using such an approach over 3 months without significant additional medication burden [58]. Whilst the resources necessary to deploy this render it likely unachievable in most clinical settings, it supports the concept that a targeted approach to correcting pathophysiology is beneficial.

Signs of a cardiac-driven hypertension may include high HR and/or ISH, especially in the context of obesity or anxiety, conditions associated with increased SNS activity. CCB agents act by causing vasodilatation, associated with a reflex tachycardia which may worsen the initial haemodynamic abnormality of high HR. A β-adrenergic antagonist (β-blocker) would be a logical agent to use to counter the specific haemodynamic abnormality of high HR or high SV in these patients. A pilot study in adults evaluated the effects on BP and HR with dual α and β blockade in a group of obese compared to lean hypertensive patients. Following one month of therapy, the obese group demonstrated a larger drop in SBP and MAP but not DBP. HR was significantly higher in the obese patients prior to treatment, and this difference resolved post-treatment [59]. Interestingly, the most recent adult 2023 ESH  has reinstated β-blockers as equally valid first/second line treatment choices. Additionally, they suggest patients with a resting HR above 80 should be treated with a β-blocker as a first line agent due to the clear independent association between high HR and adverse cardiovascular outcomes [26, 60]. β-blockers are not currently recommended as first line agents in either the 2016 ESH or 2017 AAP guidelines for the management of hypertension in children. In the authors’ opinion, this recommendation should be reconsidered considering the clear association between inappropriately elevated HR and/or SV in the pathophysiology of hypertension in childhood.

Use of β-blockers should be evaluated in context with their known effect on weight gain, found in adult patients to be approximately 1.2 kg (range 0.4 to 3.5 kg) over a mean 6-month treatment period [61]. β-Blockers have also been associated with decreased insulin sensitivity, largely because of the increased incidence of Type 2 diabetes mellitus (T2DM) reported in several large RCTs in the early 2000 s which included first and second generation β-blockers. These associations have not been reported with newer β-blocker agents [62].

Conversely, a vascular element contributing to hypertension may be associated with elevated DBP (i.e., SDH) or in patients in whom uncontrolled hypertension has been longstanding and therefore may have progressed to vascular remodelling. It may also be more prevalent in female patients. These patients may be more likely to benefit from a CCB, or other vascular-acting agent, e.g. α receptor antagonist, which acts to relax vascular smooth muscle and reduce SVR.

RAAS blockers have both cardiac and vascular mechanisms of action, plus additional benefits beyond their anti-hypertensive action, including increased insulin sensitivity and favourable effects on lipid profile. In clinical practice, they are probably underused in the paediatric age group due to contraindications during the initial workup for hypertension and in young women of childbearing age. However, once investigation for renovascular causes is complete, they are an ideal first-line agent in the absence of any other compelling indications, as discussed above. Given their complementary mechanism of action to counter many of the pathophysiological processes discussed, they may also be an ideal second-line agent to add. It should be noted that ACEi-induced angioedema is reported three times more commonly in those with African-Caribbean heritage than other ethnicities; therefore, an ARB should be preferentially chosen for these patients.

**Fig. 5:**
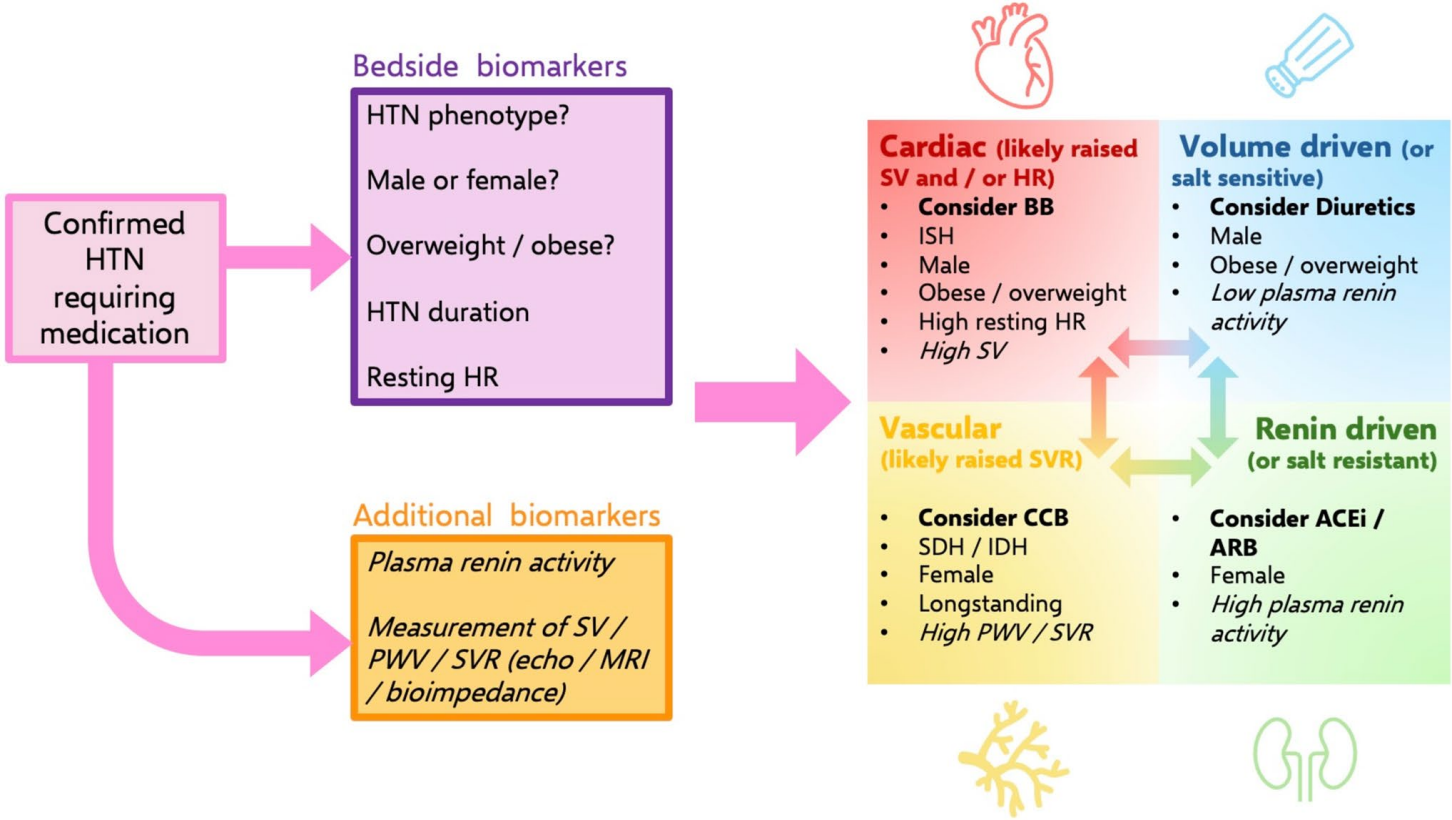
Summary of potential aetiological subtypes of primary hypertension in young people, with potential biomarkers and choice of antihypertensive agent associated with these subtypes. HTN, hypertension; HR, heart rate; SV, stroke volume; PWV, pulse wave velocity; SVR, systemic vascular resistance; MRI, magnetic resonance imaging; BB, beta blockers; ISH, isolated systolic hypertension; CCB, calcium channel blockers; SDH, systo-diastolic hypertension; ACEi, angiotensin converting enzyme inhibitors; ARB, angiotensin receptor blockers

These two presented approaches to hypertension treatment are the first steps in attempting to personalise the choice of antihypertensive agents in the young. Figure 5 summarises the different aetiological subtypes of hypertension discussed in the previous sections with potential demographics and biomarkers associated, plus a suggestion for the first antihypertensive agent in patients clearly falling into one of these categories. It should be noted that there is presumably a degree of overlap between haemodynamic and renin-based subtypes; however, the extent to which these correlate with each other has not been investigated. Obesity has been associated most strongly with the cardiac and volume-driven phenotypes; however, as discussed above, overweight patients are also at higher risk of earlier vascular remodelling. In addition, adipose tissue is known to secrete RAAS elements; thus, there are multifactorial processes contributing to obesity-related hypertension.

Clearly there is a lot of work to be done before any of these suggestions are backed by outcome data in this population. In the current era, hypertension treatment studies should go beyond the historical placebo-controlled trial or even a head-to-head comparison of two classes of medication; given the huge funding and resources required to deliver an RCT, these simple designs represent missed opportunities to gather high-quality data that truly informs clinical practice. A recent example from adult practice is the AIM-HY study, looking at optimal monotherapy and dual therapy treatment regimens in self-identifying White, Black, and South Asian patients which is due to report this year. The study used a crossover design to cycle patients through three different monotherapy or four dual therapy options and measured a number of different secondary outcomes including cardiac, vascular, and plasma-based biomarkers [63]. This design could be emulated in paediatrics comparing optimal treatment in obese vs. non-obese hypertensives, those with SDH vs. ISH, or biomarker stratified, e.g. by plasma renin activity or resting HR.

Section summary:

- There is very little evidence or direction in guidelines to use in tailoring medication options for PH in the young.
- Bedside markers with potential use in personalising choice of agent include type of hypertension (IDH, ISH or SDH), obesity status, resting heart rate and sex.
- More specialist markers with potential use include baseline plasma renin activity and cardiac haemodynamics as measured by echo or cardiac MRI (SV, aortic PWV).
- Sophisticated clinical trial designs are needed to evaluate the optimum treatment in young people with PH and the utility of these biomarkers in predicting response*.*

### Considerations of wider metabolic/cardiovascular risk

Recent longitudinal follow-up data reinforce the importance of cardiovascular risk profiling in patients presenting with PH. Identification of additional cardiovascular risk factors may not change hypertension management but will ensure timely diagnosis and management of these to alter the cardiovascular trajectory for these patients over their next few decades. There is currently no evidence to support pharmacological primary prevention strategies, e.g. statin use based on a composite cardiovascular risk score in children. Rather, hypertension, T2DM and hypercholesterolaemia should be assessed and managed individually. This may necessitate a move towards the provision of holistic cardiovascular risk management services for the paediatric population where they do not already exist. Lastly, a specific relationship between BP and hyperuricaemia has been proposed, with a small RCT demonstrating a drop in BP in hypertensive patients with hyperuricaemia following allopurinol treatment [64]. Whilst the precise mechanisms underlying this have not been fully elucidated, identification and management of hyperuricaemia should be included in the assessment for PH.

For obese and overweight young people with hypertension, weight loss should be the single most important management goal. In hypertensive and overweight young people, successful lifestyle interventions have been shown to lead to both improved BP and regression of left ventricular hypertrophy (LVH) in addition to weight loss [65]. These lifestyle interventions are notoriously difficult to implement and maintain in adolescents, but can now be supplemented in some centres with adjunctive weight loss therapies. Glucagon-like peptide 1 (GLP-1) receptor agonists have revolutionised weight management in the adult population and are starting to be used in the paediatric population following the licensing of several agents in the USA and Europe since 2020 [66]. These agents may have additive effects on BP in addition to weight and metabolic parameters. GLP-1 receptors are expressed in heart muscle and in blood vessels, and results from adult trials in Type 2 DM indicate a modest reduction in SBP of 2–3 mmHg depending on the GLP-1 agent and dose used [67]. The mechanism for this reduction remains unclear; it is in part mediated by weight loss itself; however, GLP-1 agonists have also been linked to atrial natriuretic peptide release and separately to favourable effects on endothelial and vascular function [68]. It should be noted that treatment with these agents is associated with a rise in HR (~ 3 bpm in most studies), again with an unclear underlying mechanism [67].

Section summary:

- Medication strategy for PH should be considered holistically in the context of the wider cardiovascular risk and need for concurrent lifestyle modification.
- Weight management remains the strategy with the most potential benefit to obese hypertensive patients and newer weight loss agents will likely be increasingly used soon to help achieve this.

## Conclusions

Primary hypertension in children and young people is underpinned by a variety of different pathophysiological processes. Though there is significant variation between individuals, children demonstrate a predominance of cardiac-weighted haemodynamics because of autonomic dysfunction and impaired sodium/water handling. Careful evaluation of standard clinical biomarkers, including SBP vs. DBP, HR, plasma renin, and urinary sodium excretion, may inform a more personalised approach to treatment in the absence of evidence to guide antihypertensive choice. International, multicentre randomised controlled crossover trials are urgently needed to evaluate which antihypertensive treatments are most effective in different clinical contexts and the predictive value of these biomarkers on BP response.

## Key summary points

1. Primary hypertension has different aetiological mechanisms in children as compared to older adults.
2. Isolated systolic hypertension is the predominant hypertensive phenotype in children, and is likely due to cardiac overactivity.
3. Obesity related hypertension is multifactorial involving the renin–angiotensin– aldosterone system, sympathetic nervous system and vascular elements.
4. Consideration of the duration of hypertension, hypertensive phenotype, resting heart rate and plasma renin level can give clues to underlying pathophysiology.
5. In the absence of guidelines or evidence to specify a particular agent to be used first line, selection of an antihypertensive agent should be based on likely underlying pathophysiology.

### Multiple choice questions

Answers appear following references.

1. What is the most common phenotype of primary hypertension in adolescents?
  a) Systo-diastolic
  b) Isolated systolic
  c) Isolated diastolic
  d) Isolated nocturnal
2. What is the likely haemodynamic abnormality underlying this phenotype?
  a) Increased systemic vascular resistance due to vascular ageing
  b) Increased systemic vascular resistance due to obesity
  c) Increased cardiac output due to obesity and/or dysregulated autonomic nervous system activity
  d) Increased proximal aortic stiffness due to ventricular and arterial remodelling
3. What pathophysiological mechanisms likely contribute to obesity associated hypertension in young people?
  a) Increased cardiac output due to sympathetic nervous system activation
  b) Increased systemic vascular resistance due to accelerated vascular ageing
  c) Release of renin angiotensin aldosterone system components from adipose tissue
  d) All of the above
4. Which medication is recommended first line in paediatric primary hypertension?
  a) RAAS blockade
  b) Beta blocker
  c) Calcium Channel blocker
  d) No evidence to support any agent is superior to any other
5. What biomarkers could help guide treatment in paediatric primary hypertension?
  a) Baseline plasma renin level/plasma renin activity
  b) Urine albumin:creatinine ratio
  c) Resting heart rate
  d) a) and c)

## Supplementary Information

Below is the link to the electronic supplementary material.

ESM 1 Graphical abstract (PPTX 477 KB)
