# Supplementary material for: Precision approaches to paediatric hypertension: linking pathophysiology to therapy
Source: Pediatr Nephrol. 2025 Dec 19;41(8):2393–413. doi: 10.1007/s00467-025-07100-w (PMC13337797; doi:10.1007/s00467-025-07100-w)
Supplement: Supplementary file 1 — Graphical abstract (PPTX 477 KB) [file 467_2025_7100_MOESM1_ESM.pptx]

## Slide 1
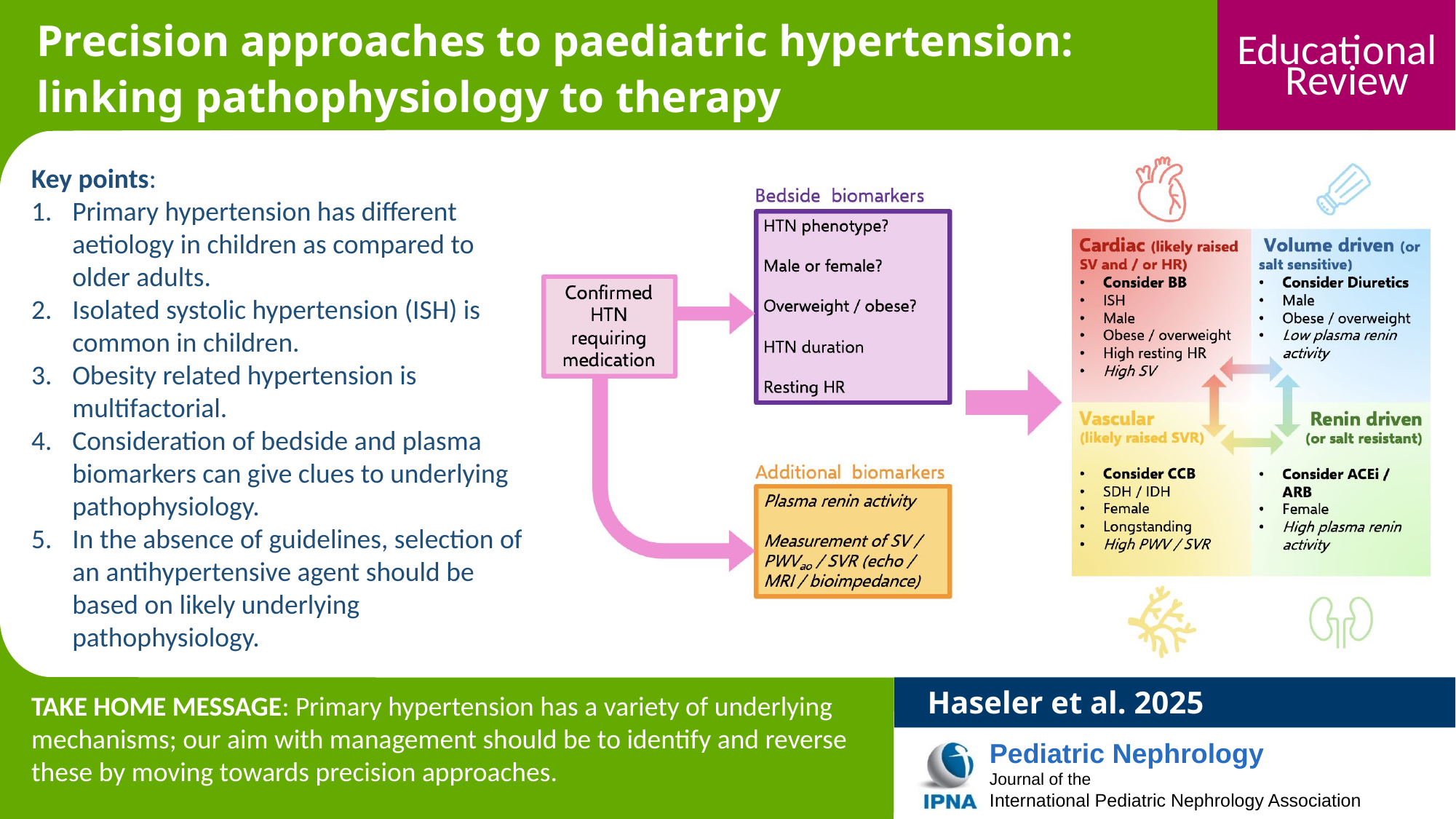

Precision approaches to paediatric hypertension:
linking pathophysiology to therapy
Key points:
Primary hypertension has different aetiology in children as compared to older adults.
Isolated systolic hypertension (ISH) is common in children.
Obesity related hypertension is multifactorial.
Consideration of bedside and plasma biomarkers can give clues to underlying pathophysiology.
In the absence of guidelines, selection of an antihypertensive agent should be based on likely underlying pathophysiology.
Haseler et al. 2025
TAKE HOME MESSAGE: Primary hypertension has a variety of underlying mechanisms; our aim with management should be to identify and reverse these by moving towards precision approaches.
